# Cardiac Arrhythmia Secondary to Loperamide Abuse and Toxicity

**DOI:** 10.7759/cureus.6936

**Published:** 2020-02-10

**Authors:** Mohammed Ali, Aisha Mujahid, Chinthaka P Bulathsinghala, Salim Surani

**Affiliations:** 1 Pulmonary and Critical Care Medicine, Corpus Christi Medical Center, Corpus Christi, USA; 2 Medicine, Shadan Institute of Medical Sciences, NTR University of Health Sciences, Hyderabad, IND; 3 Internal Medicine, Corpus Christi Medical Center, Corpus Christi, USA; 4 Internal Medicine, Texas A&M Health Science Center, Bryan, USA

**Keywords:** loperamide abuse, opioid, cardiotoxicity, qt prolongation, ventricular dysrhythmia, opioid abuse

## Abstract

Loperamide is a synthetic, over-the-counter, antidiarrheal medication that is widely available and used for the treatment of diarrhea. It is a peripherally acting opioid agonist acting mostly on the μ-opioid receptors in the gut. It was thought to be a very safe medication up until very recently, as the bioavailability of the drug is very low. At significantly higher doses, it is able to cross the blood-brain barrier and mimic the effects of centrally acting opioids. However, at these significantly high doses it also leads to significant cardiotoxic consequences. Here we present a case of a 31-year-old male with significant cardiotoxicity secondary to misuse and abuse of loperamide.

## Introduction

Loperamide is a synthetic opioid that is widely available for use as an antidiarrheal medication. It was first introduced in the United States in 1979 as a controlled substance and placed in Schedule V of the US Controlled Substances Act [1]. It was subsequently made available as a non-prescription medication in 1982 after volunteer studies demonstrated low potential risk of abuse [1]. It is an opioid receptor agonist acting specifically on the μ-opioid receptors in the myenteric plexus of the gastrointestinal tract. At therapeutic doses, it inhibits peristalsis by acting on these receptors and also exerts an antisecretory on top of the antimotility effect [2]. 

Loperamide was first synthesized in 1969; it is sold under the brand name Imodium. It was approved by the Food and Drug Administration (FDA) in 1976 for chronic diarrhea. In the 1980s, it became the best-selling antidiarrheal medication. It was briefly used in children but was withdrawn in 1990 after 18 cases of paralytic ileus were reported by the World Health Organization [3]. 

Loperamide is phenylpiperidine opioid, with a wide safety margin. It is metabolized by intestinal and hepatic cytochrome P450 CYP3A4 and CYP2C8 to inactive metabolites. Loperamide is lipophilic and highly protein bound. It has a half-life of 11 hours at therapeutic dose, but some studies have reported a half-life of up to 35 hours with overdose [1]. It undergoes biliary excretion. The elimination of loperamide is through the P-glycoprotein efflux pumps that are present in the intestinal mucosa, bile canaliculi, proximal tubule, and the blood-brain barrier (BBB). The recommended maximum daily dose of loperamide is 16 mg/day, at this dose its effects are localized to the gut. At higher does, it can have central nervous system (CNS) effects, which can be attributed to the saturation if the P-glycoprotein pumps, thereby allowing loperamide to cross the BBB and act on opioid receptors causing euphoria and analgesia [4]. Patients with P-glycoprotein polymorphism were found to have higher concentrations of the drug as opposed to patients with the wild-type gene [1,2]. Co-administration of loperamide with inhibitors CYP2C28, CYP3A4, and P-glycoprotein is associated with a fourfold increase in plasma concentration and prolonged half-life [1,4]. 

## Case presentation

**Figure 1:**
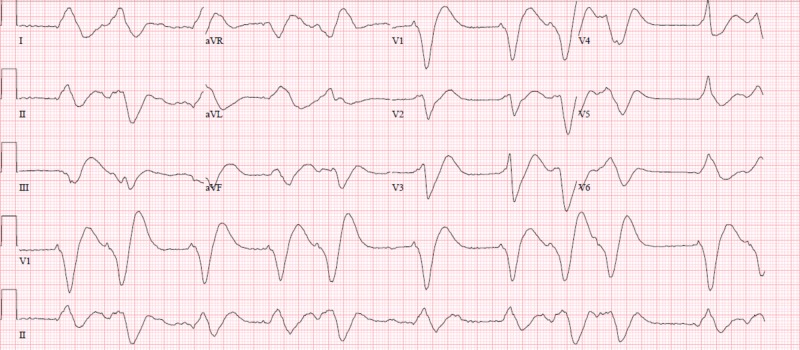
Electrocardiogram revealing wide complex ventricular dysrhythmia on presentation

**Figure 2:**
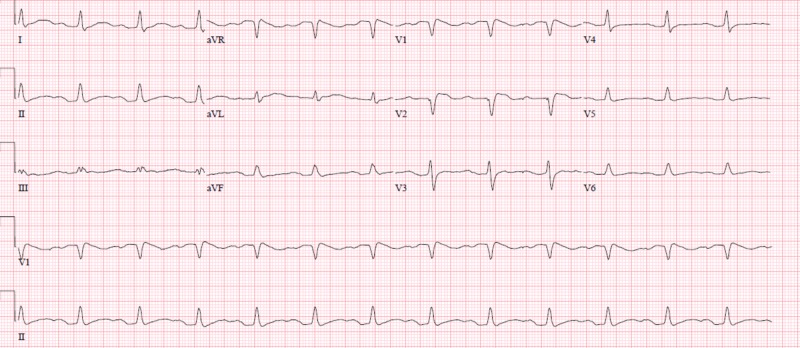
Electrocardiogram on discharge, normal sinus rhythm with prolonged QTc

A 31-year-old male with history of cystic fibrosis and pancreatic insufficiency presented to the emergency department with complaints of shortness of breath and weakness. Initial laboratory workup was significant for an acute kidney injury with an elevated creatinine at 3.03 mg/dL and mildly elevated potassium at 5.3 mmol/L with normal pH, magnesium, and calcium. Electrocardiogram (EKG) revealed a widened ventricular arrhythmia with rate varying from 25 to 85 beats per minute, short runs (<3 seconds) of ventricular tachycardia and prolonged corrected QT (QTc) of 663 milliseconds (Figure 1). He received a bolus of intravenous (IV) fluids and was started on vasopressors due to hemodynamic instability. Further questioning revealed that the patient had been consuming approximately 400 mg of loperamide daily to treat his abdominal pain and chronic diarrhea. He was managed symptomatically in addition to 2 g of IV magnesium and bicarbonate drip. Vasopressors and bicarbonate drip were discontinued after 24 hours as patient’s blood pressure returned to baseline and EKG rhythm became more regular. By hospital day 4, his EKG (Figure 2) had improved back to normal sinus rhythm with prolonged QTc and the potassium and renal function had normalized. The patient was subsequently discharged without any complications.

## Discussion

Loperamide is a synthetic opioid that is widely available for use as an antidiarrheal medication. Loperamide is not considered to have abuse potential because it is metabolized and excreted rapidly from the CNS by P-glycoprotein (a multidrug efflux pump). Its lack of CNS activity is due to minimal penetration of the BBB making it a first-line treatment for chronic diarrhea [4]. Although it is relatively safe at therapeutic doses, there have been reports of abuse recently. In 2016, the FDA released a warning about the cardiac effects causing QT interval prolongation, torsades de pointes, and cardiac arrest at higher doses [2]. 

At recommended doses, loperamide works peripherally in the gut and has very low bioavailability. When ingested in excessive amounts, it can cross the BBB and produce euphoric affects, and hence the misuse. It can also, when ingested in very excessive doses, cause cardiotoxicity. Most commonly reported cardiac manifestations are prolonged QT interval and ventricular tachyarrhythmias [5]. 

The exact mechanism of these effects is not well understood; one postulated mechanism is the blockade of the cardiac sodium/potassium channels. The mechanism of cardiotoxicity is likely related to loperamide effect on cardiac transmembrane ion channels. The QRS prolongation is most likely related to delays in depolarizations, whereas the QT prolongation is due to delays in repolarizations [4]. The human ether-a-go-go-related gene (hERG) channel is implicated in the fatal arrhythmias associated with loperamide. It pumps potassium out of the cardiac cell and plays an important role in repolarization of action potential. Loss of function of this channel is associated with hereditary long QT syndrome [5].

Management of these patients can be quite challenging, consisting mostly of supportive therapy. The exact mechanism is not very well understood and only postulated at this time. Standard overdose treatment principles can be used for loperamide toxicity. Presentation during the very early phase of ingestion is treated like any other ingestion. The use of activated charcoal is recommended during this early phase, presenting within two to four hours after ingestion [1]. If respiratory depression is observed, administration of naloxone has been suggested [6]. Loperamide toxicity is rarely the result of unintentional overdose [2]. Therefore, prompt recognition of loperamide as the offending agent can be challenging. When cardiac toxicity is suspected or encountered prompt recognition and initiation of supportive therapy is recommended. As mentioned earlier, the most common cardiac manifestations are QT prolongation and polymorphic ventricular dysrhythmias. The initiation of advanced cardiac life support is necessary in case of cardiac arrest. The use of intravenous sodium bicarbonate and magnesium sulfate has been suggested in the management of patients with ventricular dysrhythmias [1,6]. In the setting of significant QT prolongation associated with hemodynamic instability, the use of transcutaneous or transvenous pacing has been suggested [6]. Our treatment plan was also supportive with aggressive fluid resuscitation and intravenous sodium bicarbonate infusion. Much remains to be learned about the exact cardiotoxic mechanisms of loperamide.

This case was presented at the CHEST Annual Meeting, held on October 6-10, 2018, in San Antonio, TX (Poster: Cardiovascular Disease 1. https://journal.chestnet.org/article/s0012-3692(18)31269-8/fulltext).

## Conclusions

The use of loperamide as an opioid alternative is increasing. It was thought to be a very safe medication until very recently. Following a single oral dose of 2 mg the serum levels remain less than 2 ng/mL. Therefore, large quantities of loperamide are needed to achieve the euphoric and CNS opioid effects. Majority of the few cases that have been published detailing cardiotoxicity secondary to loperamide misuse and abuse were noted to have tachyarrhythmias unlike our patient. In this case, we highlight the importance of recognizing cardiac manifestations of loperamide toxicity especially given the recent rise in the abuse and misuse of the medication. This relatively new presentation of cardiotoxicity is underappreciated and requires prompt recognition. 
